# Interaction of rat alveolar macrophages with dental composite dust

**DOI:** 10.1186/s12989-016-0174-0

**Published:** 2016-11-26

**Authors:** K. L. Van Landuyt, S. M. Cokic, C. Asbach, P. Hoet, L. Godderis, F. X. Reichl, B. Van Meerbeek, A. Vennemann, M. Wiemann

**Affiliations:** 1 KU Leuven BIOMAT, Department of Oral Health Sciences, University of Leuven & Dentistry University Hospitals Leuven, Kapucijnenvoer 7, Leuven, 3000 Belgium; 2 Institute of Energy and Environmental Technology (IUTA) e.V, BliersheimerStraße 58-60, Duisburg, 47229 Germany; 3 Research Unit Experimental Toxicology, Center for Environmental and Health Research, Department of Public Health and Primary Care, University of Leuven, Herestraat 49, Leuven, 3001 Belgium; 4 Walther-Straub-Institute of Pharmacology and Toxicology, Ludwig-Maximilians-University of Munich, Nussbaumstraße 26, Munich, 80336 Germany; 5 IBE, IBE R&D gGmbH, Mendelstraße 11, Münster, 48149 Germany

**Keywords:** Dental composite, Nanoparticle, Biocompatibility, Filler, Dust, Inhalable, Occupational, Dentist, Nano-dust, Resin, NR8383, Alveolar macrophage, Biotest

## Abstract

**Background:**

Dental composites have become the standard filling material to restore teeth, but during the placement of these restorations, high amounts of respirable composite dust (<5 μm) including many nano-sized particles may be released in the breathing zone of the patient and dental operator. Here we tested the respirable fraction of several composite particles for their cytotoxic effect using an alveolar macrophage model system.

**​Methods:**

Composite dust was generated following a clinical protocol, and the dust particles were collected under sterile circumstances. Dust was dispersed in fluid, and 5-μm-filtered to enrich the respirable fractions. Quartz DQ12 and corundum were used as positive and negative control, respectively. Four concentrations (22.5 μg/ml, 45 μg/ml, 90 μg/ml and 180 μg/ml) were applied to NR8383 alveolar macrophages. Light and electron microscopy were used for subcellular localization of particles. Culture supernatants were tested for release of lactate dehydrogenase, glucuronidase, TNF-α, and H_2_O_2_.

**Results:**

Characterization of the suspended particles revealed numerous nano-sized particles but also many high volume particles, most of which could be removed by filtering. Even at the highest concentration (180 μg/ml), cells completely cleared settled particles from the bottom of the culture vessel. Accordingly, a mixture of nano- and micron-scaled particles was observed inside cells where they were confined to phagolysosomes. The filtered particle fractions elicited largely uniform dose-dependent responses, which were elevated compared to the control only at the highest concentration, which equaled a mean cellular dose of 120 pg/cell. A low inflammatory potential was identified due to dose-dependent release of H_2_O_2_ and TNF-α. However, compared to the positive control, the released levels of H_2_O_2_ and TNF-α were still moderate, but their release profiles depended on the type of composite.

**Conclusions:**

Alveolar macrophages are able to phagocytize respirable composite dust particle inclusive nanoparticles. Since NR8383 cells tolerate a comparatively high cell burden (60 pg/cell) of each of the five materials with minimal signs of cytotoxicity or inflammation, the toxic potential of respirable composite dust seems to be low. These results are reassuring for dental personnel, but more research is needed to characterize the actual exposure and uptake especially of the pure nano fraction.

**Electronic supplementary material:**

The online version of this article (doi:10.1186/s12989-016-0174-0) contains supplementary material, which is available to authorized users.

## Background

After polymerization, the surface of a composite restoration is usually finished with diamond burs, stones or disks to remove excess material. Once the restoration has a satisfying shape, the composite will be polished using disks and rubber points, wheels and cups. Finishing a composite restoration is by preference performed using water-cooling, but in clinical circumstances this is not always possible (for example when using disks, or due to reduced visibility) [1].

Previous research revealed that composite restorations release high amounts of very fine respirable dust during abrasive procedures in the breathing zone of the patient and dental operator [2]. Exposure measurements in a dental office confirmed that finishing and polishing composite restorations leads to spikes in the concentrations of nanoparticles [3]. Further laboratory assessment showed that composites, irrespective of their classification, mainly released sub-100 nm particles. These particles often consisted of very small pieces of composite with filler particles embedded in resin [4], but single nano-sized filler particles were also observed by transmission electron microscopy [3].

Given the fact that the use of composites in dental practice is still increasing and that composite is one of the most frequently used dental materials, toxicological assessment of composite dust seems warranted. Health concerns have namely been expressed with regard to inhalation of nanoparticles. Not only does their size imply deep and easy penetration into the lungs, their small dimensions also impart different chemical and physical properties to nanoparticles compared to particles of the same material with larger size [5]. In general, for the same mass dose, nanoparticles represent a much larger surface which makes them more reactive. Exposure of animals, most often rodents, to ambient or laboratory-generated nanoparticles consistently induced mild but noticeable inflammatory responses both in the pulmonary tract as well as in extrapulmonary organs. A common toxicologic mechanism of pro-inflammatory and oxidative-stress-related cell responses has been identified [6].

Whereas particles in the upper airways are mostly cleared through the mucociliary escalator [7], particles in the alveolar region will be phagocytized by alveolar macrophages [8]. In spite of their efficient phagocytosis capacity, it is, however, thought that inefficient phagocytosis with excessive production of inflammatory mediators may lead to sustained inflammation and eventually fibrotic changes [7]. There is so far no information available on the behavior of alveolar macrophages exposed to composite dust. In this study, phagocytosis, cytotoxicity, membrane integrity, release of a pro-inflammatory mediator and the production of reactive oxygen species (ROS) was investigated after exposure of alveolar macrophages to composite dust. The null hypothesis put forward was that composite dust can efficiently be phagocytized by alveolar macrophages and does not lead to membrane rupture with secondary release of lytic enzymes or ROS and does not have important inflammogenic properties.

## Methods

### Composite used

Five commercial composites (Gradia Direct, Z100 MP, Filtek Supreme, GrandiO and Tetric EvoCeram), including so-called nano (−hybrid) composites and conventional hybrid composites were included in this study. Their composition and manufacturer can be found in Table 1. For each composite, two sticks with size of 17.4 mm × 5.4 mm × 1.6 mm were prepared in a metal mold as per instructions of the manufacturer. Prior to polymerization, the composite was covered with a glass plate, and light-cured on each side for 20s with the Bluephase 20i (Ivoclar-Vivadent, Schaan, Liechtenstein) (intensity 1020 mW/cm^2^).

**Table 1:** Manufacturer and composition of the included composites

Composite	Manufacturer	Classification	Resin matrix	Filler	Filler loading
Filtek Supreme XTE	3M ESPE, Seefeld, Germany	Nano-composite	BisGMA, Bis-EMA, UDMA, TEGDMA	• SiO_2_ (20 nm) • Zirconia-silica clusters (0.6–1.4 μm) with primary particles of 5–20 nm	78.5 wt% 59.5 vol%
Gradia Direct	GC, Tokyo, Japan	Micro-filled hybrid composite	UDMA	• Silica prepolymerized filler (16 μm particles with 16 nm pyrogenic silica) • Silicate glass (850 nm) • Pyrogenic silica (16 nm)	73 wt% 64 vol%
GrandiO	VOCO, Cuxhaven, Germany	Nano-hybrid composite	Bis-GMA, TEGDMA	• Spherical nanoparticles of silicon dioxides (20–50 nm)	87 wt% 71.4%
Tetric EvoCeram	Ivoclar-Vivadent, Schaan, Liechtenstein	Nano-hybrid composite	Bis-GMA, UDMA, Bis-EMA	• Barium alumino silicate glass (0.6 μm) • Ytterbium trifluoride • Mixed oxide (160 nm) • Prepolymerized filler	82–83 wt% 66–67 vol%
Z100 MP	3M ESPE, St. Paul, MN, USA	Hybrid composite	BisGMA, TEGDMA	• Zirconia silica (0.01 μm to 3.5 μm)	84.5 wt% 66 vol%

*Abbreviations: Bis-EMA* ethoxylated bisphenol A glycol dimethacrylate, *BisGMA* bisphenol A diglycidyl dimethacrylate, *TEGDMA* triethylene glycol dimethacrylate, *UDMA* urethane dimethacrylate

Based on information provided by the manufacturer

### Collection and preparation of composite dust

These composite sticks were ground in a sterile jar using a dental bur and all dust was collected. Beforehand, the jar was heated at 200 ° C for at least 4 h to destroy any endotoxins, and subsequently sealed using a sterilization cloth with two slits. Through one slit, a handpiece (Kavo Intracompact handpiece, 200,000 rpm) with a diamond bur (842314014 Komet, Lemgo, Germany, grain size 100 μm) was placed, and fixed with autoclavation tape (Comply, 3 M, St-Paul, MN, USA). The other slit was covered with tape until cutting the composite. Next, the jar with fixed handpiece was steam-autoclaved. Prior to cutting, the composite was disinfected shortly (5 s) in ethanol (70% v/v ethanol, Hydral 70, VWR, Haasrode, Belgium). Using tweezers, the composite stick was introduced in the jar through the second slit, and after connecting the handpiece to an electric micromotor (EWLK9, Kavo, Biberach, Germany), it was ground to produce composite dust.

### Preparation of particle suspension

Particles were used either as collected, or further fractionated by sieving to remove larger, non-respirable particles. In the latter case, 1–2 mg of each composite dust was suspended in sterile double distilled H_2_O and ultrasonicated with a probe adjusted to 50 W (VibraCell™, Sonics & Materials, Danbury, CT, USA) for 10 s. The aqueous suspensions were then passed through a 5 μm filter (Partec, Görlitz, Germany) and adequate volumes (0.5 ml) were dried on pre-weighted glass cover slips (24 × 24 mm^2^). The increments in weight (three measurements) were measured with a micro balance (AT-20, Mettler Toledo, Gießen, Germany) and the mean value from each gravimetric measurement was used to adjust the particle suspension to 360 μg/ml. Finally an equal volume of double concentrated Ham’s F-12 K medium (Gibco Life Technology, Germany) or double concentrated KRPG buffer (final concentrations (in mM): NaCl (129), KCl (4,86), CaCl_2_ (1.22), NaH_2_PO_4_ (15.8), glucose (5.5 mM), pH 7.3–7.4) was added to generate cell-culture-compatible particle suspensions. The F-12 K medium used for testing was supplemented with 2 mM glutamine, 100 U penicillin and 100 μg streptomycin. Serum was not added to the medium to avoid the formation of a protein corona, which could modify the properties of the particles [9]. All media and chemicals used were from Sigma-Aldrich (Taufheim, Germany).

As negative and positive control, suspensions of corundum particles (Elektrokorund, ESK Elektroschmelzwerk Kempten, Germany) and quartz DQ12 particles (DMT, Essen, Germany) were prepared directly in F-12 K medium or KRPG [10, 11], ultrasonicated for 10 s and serially diluted as described above. In a first series of experiments, abrasion particles were used non-filtered and prepared in the same way. All critical steps of particle suspension preparation were carried out in a laminar flow bench.

### Characterization of the particles

Particles suspensions were prepared with brief ultrasonication in H_2_O and F-12 K as described above. Size measurements of unfiltered or filtered particles in H_​2_O and F-12 K medium prior to application were carried out using a Coulter counter LS230 (Beckmann Coulter, Krefeld, Germany) covering a particle size range from 0.04 μm to 2 mm. The size of nano-scaled composite particles was also determined under cell culture conditions by particle tracking analysis. Particles were dispersed in H_2_O, KRPG, or F-12 K medium (180 μg/ml) and incubated for 16 h at 37 °C in a 96 well plate. Supernatants were retrieved and pipetted onto the stage of a NanoSight LM10 instrument. The hydrodynamic diameter of particles was measured using NTA Software 2.3.

The intensity weighted hydrodynamic particle diameter and the zeta potential of the unfiltered composite dust particles were measured previously using a Delsa Nano C system (Beckman Coulter, Fullerton, CA, USA) which allows for dynamic light scattering (DLS) and electrophoretic light scattering (ELS) measurements of particles up to several micrometers [12].

### Macrophage cell culture

Rat alveolar macrophages (NR8383, ATCC: CRL-2192) were cultured according to the recommendations of the American Type Culture Collection (ATCC) in Ham’s F-12 K medium, supplemented with 100 U penicillin, 10 μg/ml streptomycin (Gibco) and 15% fetal calf serum (PAA, Cölbe, Germany) and kept under cell culture conditions (37°C, 5% CO_2_) [13, 14].

For testing purposes, macrophages were mechanically harvested from the bottom of the culture flask by gentle shaking and seeded in a 96-well microtiter plate at a density of 3x10^5^cells per well [9]. As such, the four tested concentrations could be converted into a mean dose per cell which calculates to 15, 30, 60, and 120 pg/macrophage.

After 24 h incubation at 37°C and 5% CO_2_ in a humidified incubator, medium was replaced by 200 μl of the different particle containing suspensions prepared in Ham’s F-12-K medium or with 200 μl of particle free medium (control). Cell-free wells were included to control for optical interference of particles with dyes and light scattering properties. After 16 h of treatment, the cell-free supernatant was retrieved for further analysis, centrifuged to remove cells and debris (10 min 200 xg) and distributed to various colorimetric tests (see 4–6), all of which were carried out with a microplate reader (Tecan Infinite F200 Pro, Männedorf, Germany).

### Viability assessment

The viability of particle-treated NR8383 cells was assessed through the WST-1 assay (Roche, Penzberg, Germany). This assay is based on the extracellular cleavage of the tetrazolium salt WST-1 to a soluble formazan by viable cells. After withdrawal of the medium at the end of the 16 h incubation period, 100 μl of WST-1 containing medium was added to the cells and incubated for 10 min at 37°C. Optical quantification of formazan was performed spectrophotometrically according to the manufacturer’s instruction at 450 nm and 595 nm. Cell-free particle controls were run in parallel. The mean viability was calculated by expressing values as a percentage of the unrestrained controls.

### Membrane integrity

The effect of composite dust particles on membrane integrity was assessed through two independent approaches, i.e., the lactate dehydrogenase (LDH) assay and the β-glucuronidase assay. The LDH assay assesses the membrane integrity by determination of the extracellular leakage of the membrane-impermeable cytoplasmatic enzyme LDH. The lysosomal enzyme β-glucuronidase is actively secreted from activated alveolar macrophages and/or is released from deteriorated phagolysosomes, e.g. if phagocytosis is disturbed or cells undergo necrosis. For the assays, centrifuged cell culture supernatant (50 μl) was incubated with 50 μl LDH reaction mix (Cytotoxicity Kit, Roche) as described by the manufacturer. The reaction was stopped with 1 N HCl and optical density (OD) was measured at 492 and 620 nm (reference value). Values were corrected for cell free-adsorption and normalized to the positive control value (set to 100%) obtained from non-treated cells with 0.1% triton X100. To measure glucuronidase activity, 50 μl of the supernatant were incubated with the reaction mix containing p-nitrophenyl-D-glucuronide (13.3 mM), 0.1% Triton X-100 dissolved in 0.2 M sodium acetate buffer (pH 5) for 2 h [10]. The reaction was stopped after 2 h with 100 ml 0.2 N NaOH. Optical density was measured at 405 nm; values were corrected for cell free-adsorption and normalized to a positive control value (100%) obtained by lysing non-treated cells with 0.1% triton X-100.

### Cytokine release and cytolysis assay with L-929 cells

Supernatants of particle-treated and control cells were analyzed for bioactive TNF-α using the L-929 cytolysis test, in which the number of murine fibroblasts (L-929) is reduced based on the TNF-α concentration present in the supernatant [15]. In brief, 50 μl centrifuged supernatant from each well was pipetted onto confluent L-929 cells in the presence of actinomycin D. After 24 h cells were stained with 0.5% crystal violet. Then cells were washed with phosphate buffered saline and lysed in an acidic mixture of citrate buffered 50% ethanol [15]. Optical density was measured at 570 nm. Results were expressed as per cent lysis of L-929 fibroblasts relative to non-treated controls. In all tests the TNF-α forming capacity of NR8383 cell was functionally controlled by stimulation with lipopolysaccharide (0.1 μg/ml) and by quartz DQ12-treatment. Responsiveness of L-929 cells to TNF-α was also controlled with a TNF-α standard (R&D 510-RT, Bio-Techne GmbH, Wiesbaden-Nordstadt, Germany). To exclude effects of particles transferred from NR8383 culture onto L-929 cells, supernatants from cell-free particle controls were run in parallel, but no effects could be observed. Results were expressed as per cent lysis relative to control.

To measure Il-1β, undiluted supernatants of particle-treated and control NR8383 cells were evaluated using an Enzyme Linked Immunosorbent assay specifically detecting rat IL-1β (ELISA, Bio-Techne GmbH, Wiesbaden-Nordenstadt, Germany).

### Determination of oxidative stress

Generation of oxidative stress was analyzed using the Amplex Red assay. Quantification is based on the formation of resorufin which gives a quantitative i.e. stoichiometric measure of H_2_O_2_ concentration. All chemicals used for this test were purchased from Sigma-Aldrich. NR8383 cells were seeded in a 96-well microtiter plate at a density of 3 × 10^5^ cells per well and incubated under cell culture conditions for 24 h. The medium was then replaced by 100 μl of the particle containing suspensions prepared in KRPG or by KRPG alone (control). Immediately thereafter, 100 μl/well of a freshly prepared reaction mix containing Ampliflu (0.1 mM), NaN_3_ (2 mM), and horseradish peroxidase (2 U/ml) was added and incubated under cell culture conditions for 90 min. Zymosan (360 μg/ml) was used as a positive control [16]. Accuracy of the reaction was controlled with a H_2_O_2_ standard concentration (30 μM) freshly prepared from a 30% H_2_O_2_ stock solution. OD was measured at 570 nm and 620 nm (reference value), corrected for background absorbance of cell free-particle controls, and converted into absolute concentrations of H_2_O_2_ using the molar extinction coefficient of resorufin (54,000 L x mol^−1^ x cm^−1^).

### Uptake of particles by macrophages

The sedimentation and uptake of composite dust particles by the macrophages through phagocytosis was evaluated by light microscopy combined with particle tracking technology. Sedimented particles were viewed at the bottom of the 96 well plate with an inverted phase contrast microscope (Zeiss Axiovert40C) equipped with a CCD camera (ICC3) to document the uptake of particles. Particle tracking analyses were carried out on cell culture fluid at the end of the testing period using a NanoSightLM10 instrument (Malvern Instruments, Malvern, UK) equipped with a green laser (532 nm) and NTA 2.2 software. This combined approach allowed to detect gravitationally settled single or agglomerated particles down to the range of 0.5 μm, while the Nanosight technique detects nanoparticles down to approximately 30 nm.

Additionally, the uptake of the particles was analyzed by transmission electron microscopy (TEM). NR8383 cells were cultured on sterile Aclar or Melinex foil (Plano, Wetzlar, Germany) sterilized in 80% ethanol and incubated with 90 μg particle dust per ml F-12 K medium (filtered fraction of particles) for 16 h. Thereafter, the medium was replaced and cells were fixed in 2.5% glutaraldehyde in 0.1 M sodium phosphate buffer (pH 7.3) for 60 min. Samples were washed 3 times with the same buffer, post-fixed in 1% OsO4 and dehydrated in ascending concentrations of ethanol. Staining with uranium acetate (1%) was carried out at the 70% ethanol step. After further dehydration with ethanol and propylene oxide, foils with samples were mounted in appropriate moulds and embedded in Epon 812 (Sigma Aldrich, Taufkirchen, Germany). Sections of 60–90 nm were obtained by ultra-microtomy using a diamond knife and analysis was carried out by TEM (JEOL, JEM-1200 EX II Tokyo, Japan) at a voltage of 80 kV.

### Statistical analysis

All quantitative data are presented as mean ± standard error of the mean. All biological assays were performed in three independent experiments with triplicate cell cultures. Dunnett’s multiple comparisons test (one-way ANOVA) was used to test for biologic effects of particles. A p-value of <0.05 was considered statistically significant. To compare dose-dependent effects between the treatment groups, Tukey’s multiple comparison test was used. All statistical calculations were carried out with GraphPad Prism software (version 5.01).

## Results

### Particle characterization

**Fig. 1:**
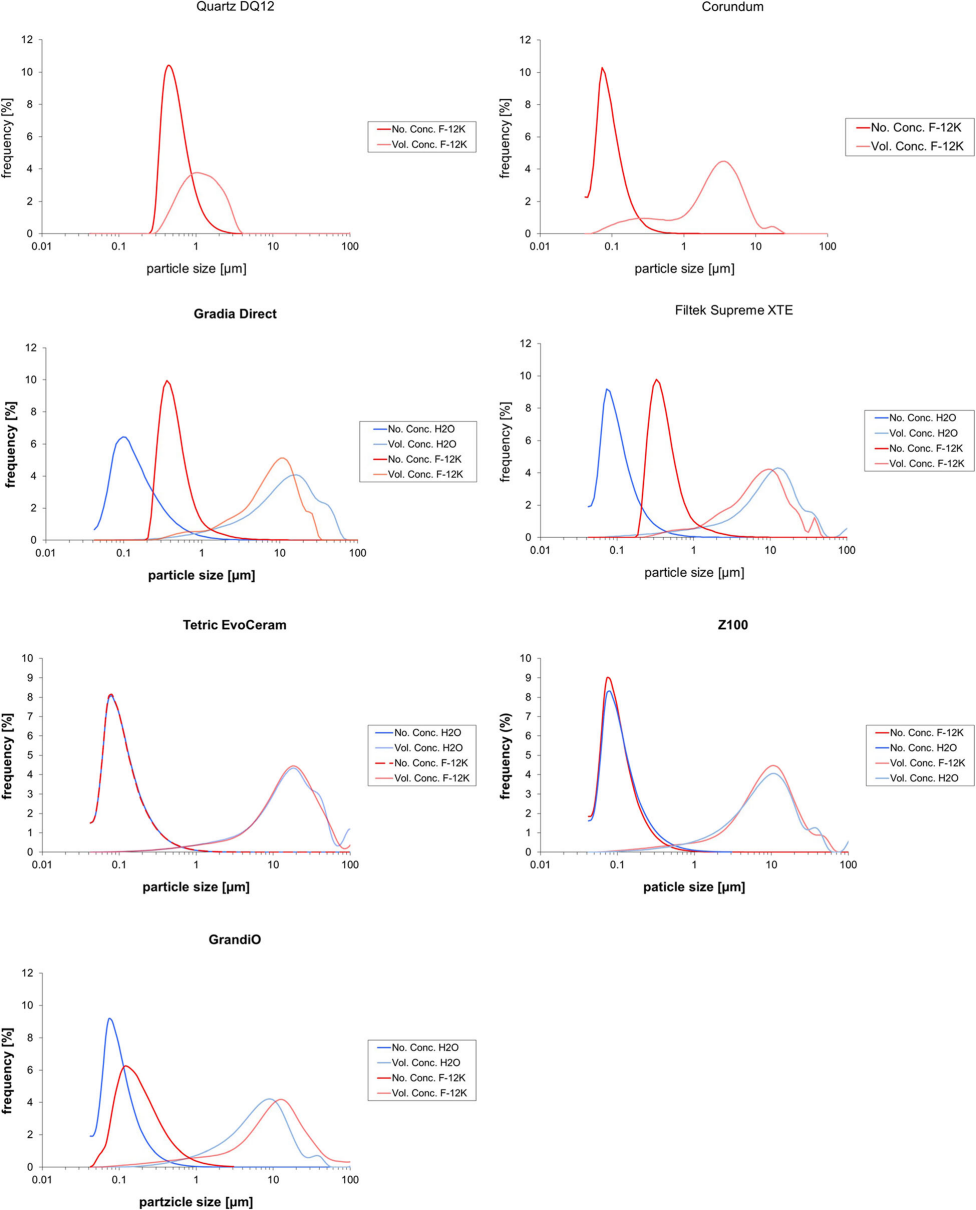
Particle size distribution of unfiltered particles in H_2_O and cell culture medium. Coulter Counter analysis showing number concentration (No. Conc.) and volume concentration (Vol. Conc.) of particles dispersed in F-12 K (corundum, Quartz DQ12, composite particles) and for double destilled H_2_O (composite particles only). Note that size maxima shift of the number concentration peaks shift to larger values except for Z100 MP and Tetric EvoCeram

The size distribution of the original unfiltered particle preparation in H_2_O and also in cell culture medium is shown in Fig. 1. Although the volume concentration curves show a considerable amount of high volume particles, all composite particles contained numerous nano-sized particles indicated by the peaks of the number concentration curves. It is also obvious from Fig. 1 that the number concentration peaks of Filtek Supreme, Gradia and GrandiO were shifted to higher values indicating agglomeration in F-12 K medium, i.e., under cell culture conditions. In contrast, the size distribution of Tetric EvoCeram and Z100 MP in H_2_O and medium remained nearly unaltered.

**Fig. 2:**
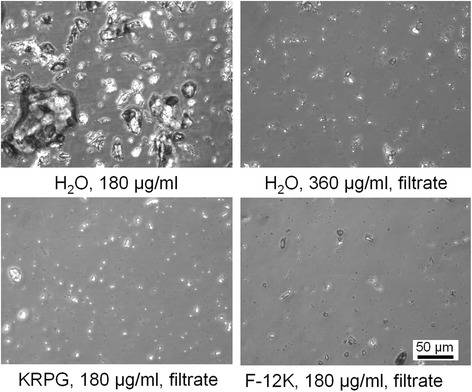
Effect of filtering on particle size. Unfiltered particles (Gradia Direct, *upper left*) dispersed in H_2_O were passed through a 5 μm cell strainer, which eliminates the dominant, non-respirable particle fraction (*upper right*). Resulting particles were mixed with double concentrated KRPG (*lower left*) or double concentrated F-12 K medium (*lower right*) to establish cell culture conditions. Phase contrast micrographs taken from the bottom of a 96-well plate after complete sedimentation

To eliminate the large non-respirable particles from the suspension for the purpose of biological in-vitro testing, the aqueous suspension was filtered through a 5 μm cell strainer. Figure 2 shows that the resulting sub-fractions, after mixing with double concentrated KRPG buffer or F-12 K medium, were completely devoid of large particles while smaller particles appeared homogeneously distributed. The occurrence and size distribution of nanoparticles was not impaired by this process as measured by coulter counter, and the curves of filtered and non-filtered composite particles were largely congruent (Fig. 1). To test if nanoparticles prepared by filtering remain in the cell culture fluid until the end of the cell culture period, cell culture supernatant was subjected to tracking analysis. Table 2 shows that nanoparticles, albeit measurable in H_2_O, were reduced in number in the F-12 K medium under cell culture conditions, which is in line with coulter counter analysis of the unfiltered suspensions shown in Fig. 1. As in that analysis, however, few nanoparticles of Filtek Supreme, Tetric EvoCeram and Z100 MP remained in F-12 K medium under cell culture conditions.

**Table 2:** Particle size of filtered composite dust particles in H_2_O and under *in-vitro* conditions as measured by tracking analysis

	Filtek	Gradia	Tetric	GrandiO	Z100
H_2_O					
D50 (nm)	214	209	165	154	168
Conc. [x10^8^/ml]	3.66	4.56	1.28	3.32	3.99
F-12 K Medium					
D50 (nm)	n.m.	n.m.	124	n.m.	172
Conc. [x10^8^/ml]	n.m.	n.m.	0.12	n.m.	1.43

*Abbreviations: D50* Mass-median-diameter (MMD), *n.m.* not measurable due to low particle number

DLS measurements (previous research) showed that the mean particle diameter or so-called Z-Average, which is the harmonic intensity averaged particle diameter, varied between 1.35 and 4.12 μm (Table 3). The polydispersity index (PI), which is a measure for the homogeneity of the particle size, for all composites was well below 0.7, indicating that the variability in size was not too high and that DLS analysis was a suitable means to evaluate the particles in suspension. With a PI of 0.045, the distribution of the dust particles of Gradia Direct was highly monodisperse, while Tetric EvoCeram had the broadest particle size distribution. Considering that a Zeta potential between −10 and +10 mV is considered approximately neutral, the Zeta potential of composite dust particles determined by ELS was slightly negative for all particles (Table 3) [8].

**Table 3:** DLS and ELS characterization of unfiltered composite dust in the cell culturing medium

	Z - average (nm)	SD	PI	D10 (nm)	D50 (nm)	D90 (nm)	Zetapotential mV
Gradia Direct^a^	1803.5	110.6	0.045	1354.1	1760.8	2303.7	−15.92
Z100 MP^a^	2749	391	0.085	1719	2799	4703	−14.35
Filter Supreme XTE^a^	4129	940	0.101	2345	3027	4035	−15,84
Tetric Evoceram^a^	1354.3	42.8	0.186	839.4	1399.4	2357.4	−25.52
Grandio^b^	2088.5	72.8	0.174	1347.2	1998.1	2912.4	−18.84

*Abbreviations: SD* standard deviation, *PI* polydispersity index, *D10* percentile 10, *D50* median, *D90* percentile 90

^a^Concentration of 3.3 mg/ml; ^b^concentration 0.6 mg/ml

Results from previous research (accepted for publication in Dental Materials 2016 [12])

### Cell culture testing

**Fig. 3:**
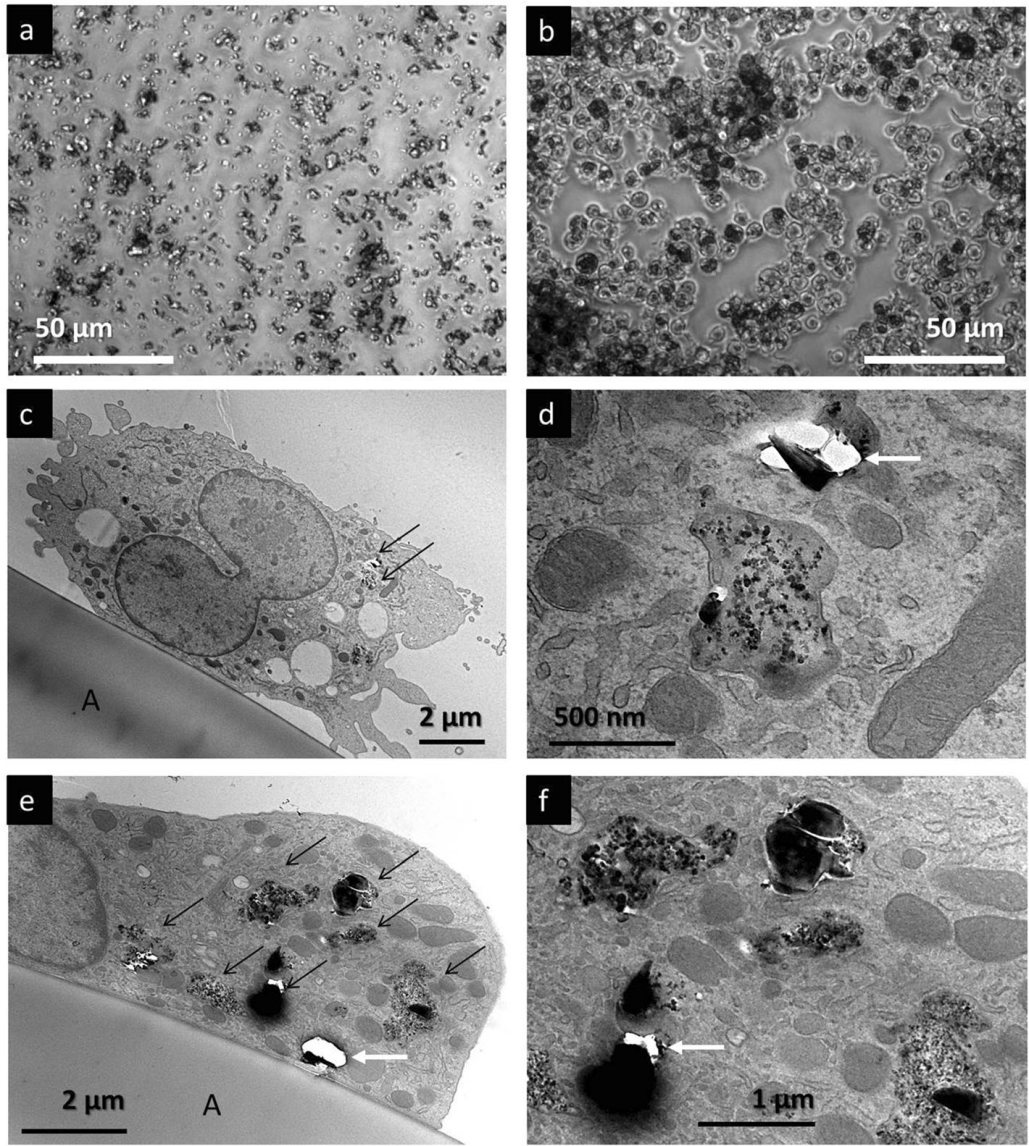
Uptake of composite dusts by NR8383 macrophages in F-12 K medium. All photomicrographs were taken after 16 h in culture. **a** and **b** represent light microscopic images, while **c**, **d**, **e** and **f** are transmission electron microscopic images. **a**: particles settled under cell-free culture conditions at the bottom of the culture vessel. Here GrandiO is shown, but the observations for the other particles were very similar. **b**: The sedimented particles as shown in **a** have been taken up by macrophages. Note that the space between the cells has been cleared from particles and most macrophages appear dark due to particle uptake, indicating that the macrophages have ingested the entire dose. The observations for the other composites were very similar. **c**, **d**, **e** and **f**: Transmission electron microscopy indeed confirmed that the particles were phagocytized by the macrophages. **c** and **e**: Macrophages on the aclar film (A). In the cytoplasm, vesicles, vacuolae, mitochondriae and endoplasmatic reticulum can be discerned. Also several phagosomes with internalized composite dust particles can be observed (*black arrows*) in the cytoplasm. **d** and **f** show a detail of **c** and **e**, illustrating the engulfed particles in the phagosomes. Dust particles containing nano-sized filler particles can be observed, but also large silicate glass particles have been taken up. As cutting these glass particles by ultramicrotomy is difficult (even with a diamond knife), this often results in artifacts due to particles that have fallen out of the section (*white arrows*). The membrane of the phagosomes can clearly be recognized

Phase contrast microscopy showed that all filtered particles in this study underwent rapid sedimentation during the incubation time and no major differences were noted between the five composite particles (Fig. 3a and Additional file 1: Figure S1). As NR8383 cells are actively moving phagocytes and covered more than 60% of the bottom area, they completely engulfed all qualities of the fine composite dust. Therefore, no particles remained detectable between the darkened particle-laden cells at the end of the culture period (Fig. 3b). Nanosight measurement of the supernatant further revealed that the uptake of particles was complete, except for the negligible number (<1×10^8^) of Z100 MP and Tetric EvoCeram nanoparticles which remained in the supernatant (Fig. 3a and b). TEM confirmed that the particles were phagocytized by the macrophages. Typically, multiple large phagolysosomes (0.5–5 μm) containing composite dust particles can be observed in the cytoplasma of the macrophages (Fig. 3 c, d, e and f). Some cells were completely loaded with engulfed composite dust, while others only contained 2–3 phagosomes, a finding corresponding to light microscopic observation. The engulfed dust particles typically consisted of multiple filler particles and resin matrix.

**Fig. 4:**
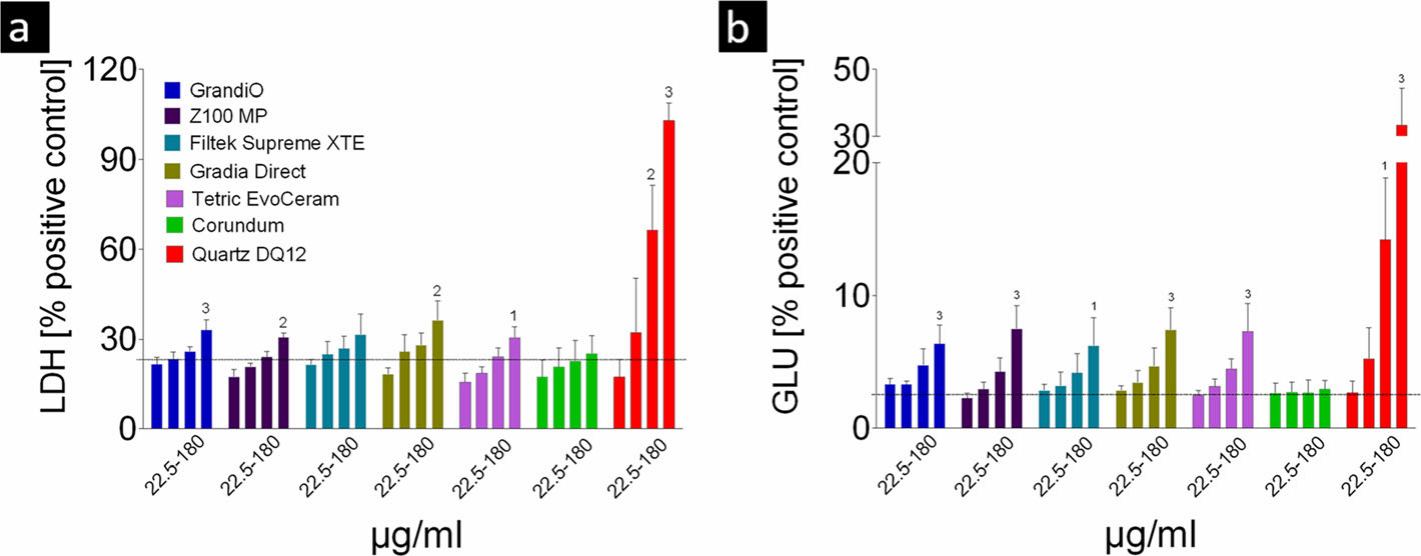
Membrane integrity of the cells was evaluated by the LDH (**a**) and the glucuronidase assay (**b**). Data are presented as mean and standard error of the mean. The concentration of filtered composite dust (X-axis) is expressed in μg/ml. The numbers above the bars indicate statistically significant differences compared to the control (1: *p* < 0.05; 2: *p* < 0.01 and 3: *p* < 0.001). The *black dotted line* in each diagram indicates the level of non-treated cell controls. Only the highest concentration of 180 μg/ml induced a significant increase in LDH and glucuronidase release. The relatively high values of 20–25% LDH (relative to the 0.1% Triton-X100 positive control, which releases all LDH from the cells) in the absence of particles are normal for NR8383 macrophages. As for the glucuronidase assay, only very mild effects of the treatment with composite dust can be observed when compared to the positive control (Quartz DQ12)

Exposure of alveolar macrophages to the unfiltered fraction, which encompassed very large particles of composite dust (Fig. 2), had no effects on viability (WST-1), LDH, glucuronidase, TNF-α or generation of ROS (data not shown). However, exposure to the same mass of the fine filtered fractions resulted in dose-dependent effects, at least upon the high concentrations induced responses. Only slightly increased levels of LDH and glucuronidase could be observed for all composites (180 μg/ml) compared to the positive control with quartz DQ12 (Fig. 4a and b). Direct comparison between the five composites revealed minor though significant differences in the release of LDH, but not in the release of glucuronidase. Tetric EvoCeram resulted significantly less LDH release than Gradia Direct, Z100 MP and GrandiO. There was also a significant difference in LDH release between Gradia Direct and Z100 MP.

**Fig. 5:**
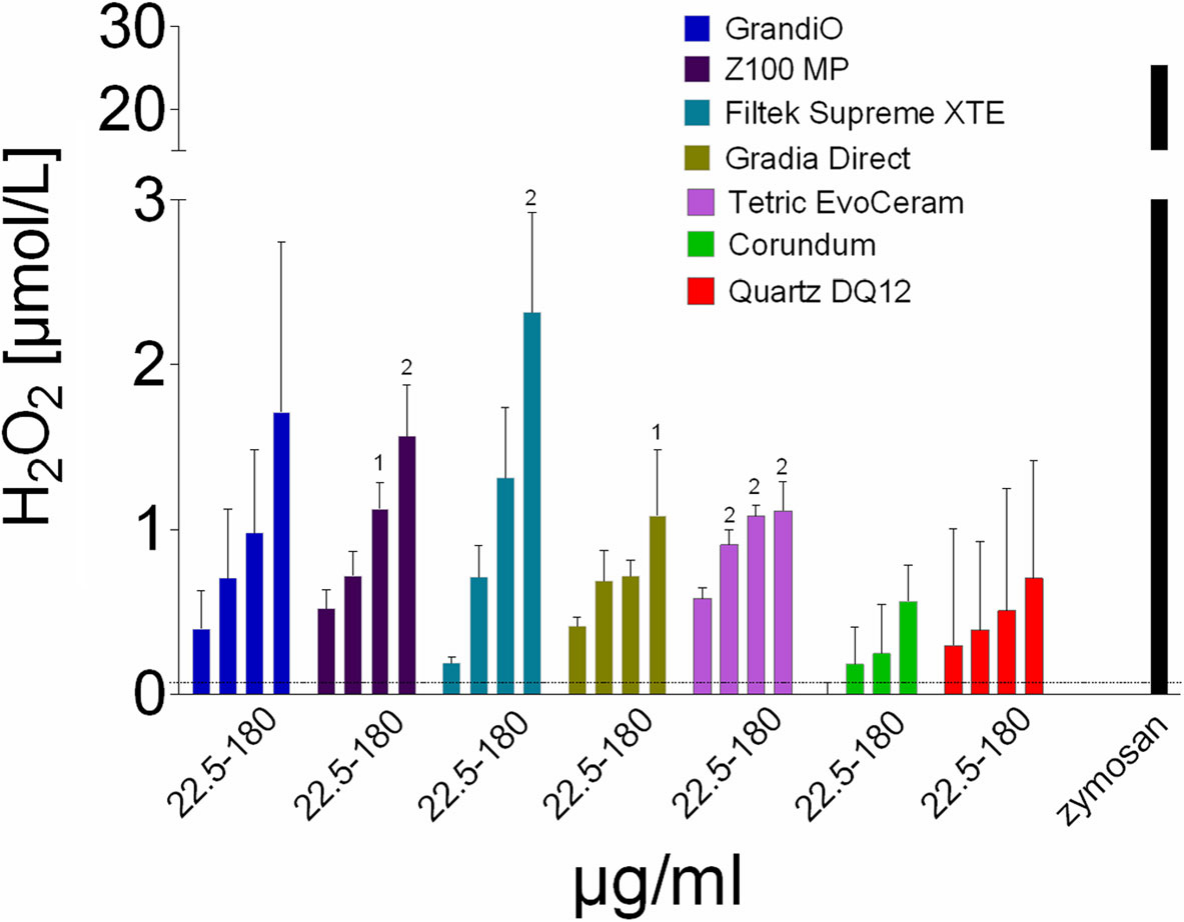
The results of the Amplex Red assay to detect generation of ROS. Data are presented as mean and standard error of the mean. The concentration of filtered composite dust (X-axis) is expressed in μg/ml. The numbers above the bars indicate statistically significant differences compared to the control (1: *p* < 0.05; 2: *p* < 0.01 and 3: *p* < 0.001). The *black dotted line* in each diagram indicates the level of non-treated cell controls. Only the highest concentrations of composite dust increased the generation of ROS, especially for GrandiO, Z100 and Filtek Supreme. However, compared to the positive control with Zymosan, which was included as a measure to indicate the capability of the cells to generate H_2_O_2_ as DQ12 quartz is not a positive control in this test, the release after composite-dust exposure was still more than 10 times lower

With regard to H_2_O_2_ release, a mild induction compared to the control was noticed especially for GrandiO, Z100 MP and Filtek Supreme (Fig. 5). This increase in H_2_O_2_ release was, however, not statistically significant. Exposure to zymosan elicited a high rate of H_2_O_2_ release indicating the macrophages were highly capable of generating a respiratory burst, for which DQ12 is not a positive control. Compared to the positive control, H_2_O_2_ release after exposure to composite dust was, however, still 10 times lower than after exposure to zymosan.

**Fig. 6:**
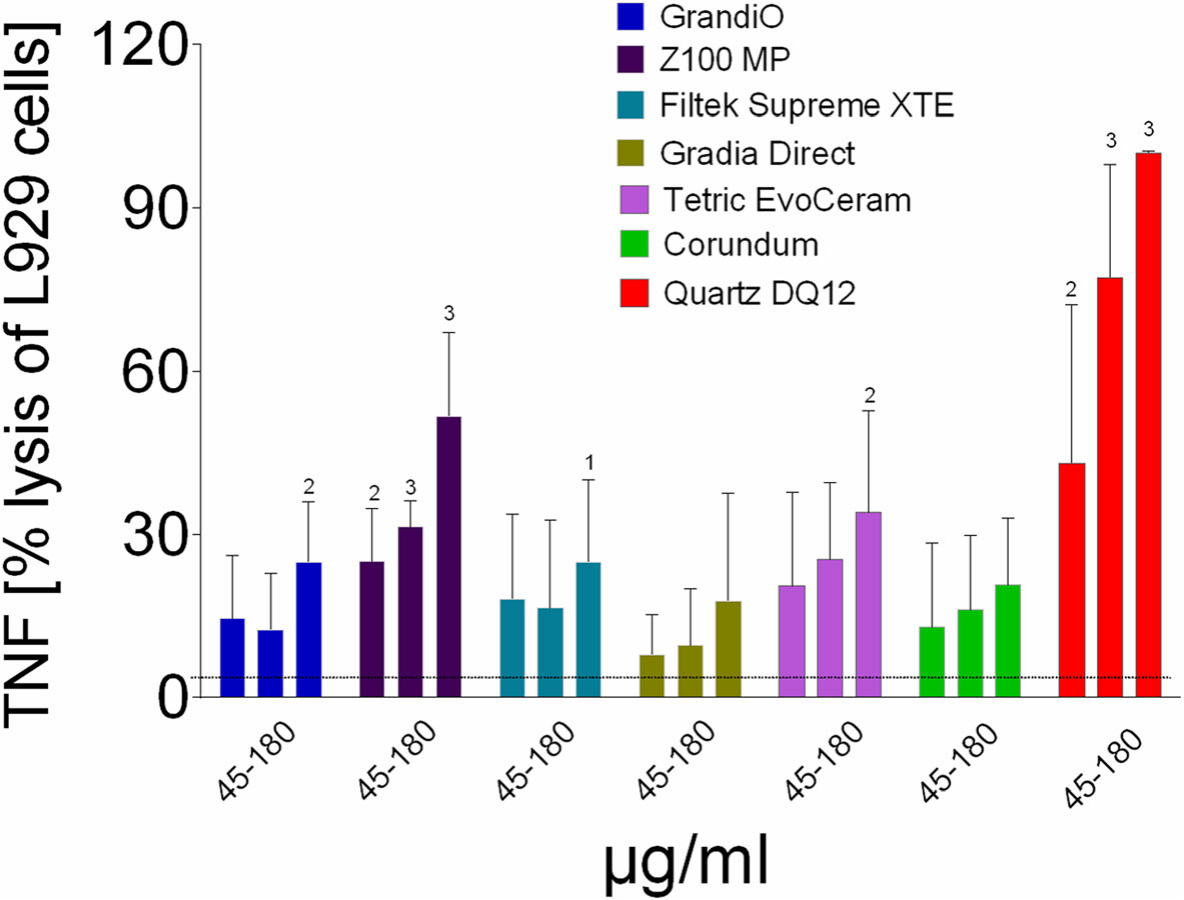
Cytolysis of L-929 cells after exposure to supernatans of NR8383 macrophages exposed to the respirable fraction of composite dust, indicating the release of TNF-α. Data are presented as mean and standard error of the mean. The concentration of filtered composite dust (X-axis) is expressed in μg/ml. The numbers above the bars indicate statistically significant difference compared to the control (1: *p* < 0.05; 2: *p* < 0.01 and 3: *p* < 0.001). The *black dotted line* in each diagram indicates the level of non-treated cell controls. L-929 fibroblasts subjected to N8383 macrophage supernatant underwent apoptosis at high concentrations (180 μg/ml) of GrandiO, Z100MP Filtek Supreme and Tetric EvoCeram, indicating release of bioactive TNF-α

L-929 fibroblasts subjected to NR8383 macrophage supernatant showed significantly increased cell death upon exposure to Z100 MT (>45 μg/ml) of GrandiO, Filtek Supreme (>180 μg/ml), and TetricEvoCeram (>180 μg/ml), indicating release of bioactive TNF-α (Fig. 6). Z100 MP and Tetric EvoCeram elicited the largest responses. Statistical analysis showed that Z100 MP induced significantly more cytolysis than Filtek Supreme, Gradia Direct and GrandiO. There also was a significant difference between Tetric EvoCeram and Gradia Direct.

Increased release of IL-1β was observed for quartz DQ12 (90 μg/ml elicited 43 ± 2.6 pg/ml) or lipopolysaccharide (0.1 mg/ml elicited 214.5 ± 4.5 pg/ml). Exposure to composite dust, however, did not result in detectable increased IL-1β release (results not shown).

## Discussion

### General

Previous research revealed that airborne composite dust consists mainly of very small particles with nano-scale sizes that may typically be deposited in the alveolar region. In this region of the lungs, alveolar macrophages represent the main non-specific host defence mechanism against exogenous particles. Besides regulating the immune response through release of many secretory products and interacting with other cells through the expression of different surface receptors, alveolar macrophages can remove foreign particles from the alveolar surface through phagocytosis, a process which does not exclude nano-sized objects [17]. Even though this macrophage response is desirable, it is also important to note that particle laden macrophages may trigger an exaggerated inflammatory response, which in case of e.g. quartz particles leads to acute pulmonary injury and develops into chronic pathology [18, 19]. As cultured alveolar macrophages (NR8383 cells) show many of their specific responses also *in vitro* [8, 11, 13, 14], we used these cells and exposed them to dust particles prepared from different types of composites. The most striking finding of this investigation, which could be considered as a first screening for potential toxic effects, is that abraded particle preparations from dental composites, which contain considerable amounts of nanoparticles [3], inferred virtually no cytotoxicity to cultured alveolar macrophages *in vitro* up to a cell burden of 60 pg/cell. This outcome indicates that the macrophage response upon composite dust strongly resembles that upon corundum particles, which were used as a negative control material as they have no toxic effect on the lung unless overload doses are applied [19–21].

### Methodological considerations, particle dispersion and cellular dose

Five different types of commercial composites were included in this study in order to be able to generalize the outcome of this study, and composite dust was generated in a clinically relevant way by cutting polymerized composite sticks with a diamond bur typically used in dental practice to remove old composite restorations, or to remove excess composite material. Care was taken to collect all particles including the very small ones, and microbial or endotoxin contamination could be excluded due to sterile handling of the sample. Our first in-vitro tests with the original, unfiltered composite dusts did not result in any particular effect, but these preparations included many large particles (>10 μm) which were neither respirable nor could they be engulfed by macrophages in-vitro. Therefore, a respirable fraction (<5 μm) of composite dust was prepared by filtering an aqueous suspension through a 5 μm cell strainer. This method was found very helpful for investigating limited amounts of powdered samples, as it allowed to gravimetrically determine filtered yields and preserved nanoparticles in a well dispersed state. Coulter Counter analysis of filtered particles furthermore showed that size distribution of all composite particles hardly differed in H_2_O and F-12 K medium, demonstrating that cells were indeed exposed to the nanosized fraction.

It might be objected that this study was unable to differentiate between the effects of nano- and micron sized particles. However, a mixed population of particles is released during the grinding process and with respect to the exposure of dental personnel and exposed patients, the analysis of the complete respirable particle fraction reflects the real life situation, whereas further isolating the nano-subfraction could have caused a considerable bias towards ultrafine particles. Nevertheless, the finding that the filtered fraction of composite dust, in contrast to the raw preparation, was more biologically active is most likely due to the larger specific surface area of smaller particles, a finding reported for other nanoparticles [22, 23].

According to our own unpublished data, a large composite stick with a volume similar to a very large composite restoration, may result in approximately 300–800 μg respirable dust, which may be found in the breathing area of the patient or the personnel [3]. If air exchange is insufficient and the volume of the breathing area is estimated to 10 L, particle concentration may transiently exceed 30 mg/m^3^. This value represents the upper range in many animal experiments and leads, after 4 weeks, to a macrophage loading of >60 pg/cell [24]. Certainly this is a worst case scenario and it is obvious that the loading of macrophages in the human lung will depend on the frequency and duration of such exposures. Nevertheless, the dose range used in this study should be regarded as relevant for the work place exposure scenario, but there is definitively a need to accurately characterize the exposure to composite dust in dental personnel. It should also be stressed that the range of particle mass per macrophage as tested here is toxicologically relevant and would have been reached in a rat experiment when a particle concentration of approximately 30 mg/m^3^ was applied over 28 days [24]. Certainly these conditions are at the upper end of typical rat inhalation studies and are presumably far away from what can be measured in a dental practice, especially taking into consideration that exposure is not continuous, but typically occurs for short instances [2, 3]. Note that the eight hour shift average occupational exposure limit for respirable dust (A-Staub) in Germany is only 1.25 mg/m^3^ [25]. Also it is important to note that particles in our in-vitro tests settled and were completely ingested by the NR8383 cells. The mean cellular load calculated under the experimental conditions therefore ranged from 15 to 120 pg per cell and this is quite close to the 90 pg measured in alveolar macrophages from rat broncho-alveolar lavage fluid after inhalation exposure [24]. It may also be calculated that a particle load of 60 pg per alveolar macrophage is roughly equivalent to a lung burden of 1.2 mg/rat lung, if it is assumed that particles are entirely contained in the macrophage compartment, and that the lung inhabits ca. 2×10^7^ alveolar macrophages [26]. Therefore, the doses applied in this *in-vitro* study to alveolar macrophages reflect an experimental scenario which is typically achieved in the course of inhalation studies on rats.

### Alveolar macrophage responses to composite material dusts

Effects of particles as tested were generally moderate and, if so, became apparent mainly at high concentrations. As some of the particles incorporated in the composites such as SiO_2_ have already been shown to be cytotoxic in vitro, moderate or missing effects may be explained by the fact that the particles were most probably still covered by resin, as observed previously by transmission electron microscopy [3, 27]. However, all composite dusts led to increased activities of LDH and glucuronidase in the supernatant which slightly outscored those of corundum particles. Interestingly, the dose dependence of the release of LDH or glucuronidase release was surprisingly similar for all particles with significance being reached at the highest concentration step only. This suggests that a damage of the cell membrane due to necrosis or apoptosis takes place only in heavily loaded cells. Obviously this process was accompanied by a release of lytic enzymes likely liberated from deteriorated lysosomes. It should be noted that a release of glucuronidase or other lytic enzymes may also happen as a consequence of macrophage activation [28], i.e., it does not necessarily demand preceding membrane damage. However, the large degree of similarity obtained in LDH and glucuronidase profiles (see Fig. 4a and b) argues against a specific activation inferred by one of the composite dusts. A high activity of lytic enzymes inside the lung parenchyma may damage the lung surfactant layer and impair lung function. However, compared to the effect of positive control quartz DQ12, no effects were obvious upon 60 pg per cell, suggesting that none of the five composite material dusts is specifically toxic for alveolar macrophages in vivo. It is therefore likely, that also in case of human exposure the deep regions of the lungs can be cleared from composite particles.

The uniform picture found for indicators of cell damage was less pronounced with respect to the induction of radical oxygen species. H_2_O_2_, whose immediate formation upon particle exposure was measured here, is mainly derived from the disproportion reaction of the O^2−^ superoxide anion which is the primary product of macrophages during the so-called oxidative burst, in which O_2_ is reduced by activating the membrane bound NAPDH oxidase. The way how particles can stimulate a respiratory burst is still incompletely understood and may involve various signalling pathways [29, 30]. Typically, a small increase in H_2_O_2_ formation occurs even upon non-toxic control particles such as corundum at high cellular dose, and this may be in line with some effects of corundum which take place in the lung under overload conditions [19]. Interestingly, the H_2_O_2_ inducing potential of GrandiO, Z100 MP and Filtek Supreme XTE, was more pronounced than that of Gradia Direct and Tetric EvoCeram (Fig. 5). At present, there is no obvious material-based explanation for differences between the composite dusts, as material composition, oxidative surface reactivity, or OH^.^ generation potential of all composites were relatively similar and data from our previous study provided no clues as to what constituent could underlie increased bioactivity [3]. Nevertheless, there may be biologically relevant differences in surface chemistry and/or structure which trigger certain effects when particles are engulfed by alveolar macrophages and it would be interesting to conduct a more detailed study on signalling pathways and release of mediators.

At present the biological significance of the released quantity of H_2_O_2_ upon composite materials can only be estimated: all responses were slightly larger than the corundum control, but by far smaller than that induced by zymosan (a yeast cell wall preparation) which fully stimulated the respiratory burst. It has been shown that similarly zymosan-stimulated NR8383 cells damaged neighbouring fibroblasts and increased their mutation rate via released radical oxygen species [16]. However, as the formation of H_2_O_2_ upon composite dust was about 15 to 30-fold lower than the positive control, similar effects emanating from composite dust laden alveolar macrophages are very unlikely, especially under in-vivo conditions.

Exposure of NR8383 cells to composite dusts also led to some cytolysis of L-929 fibroblasts, which is indicative of the formation of bioactive TNF-α. To the best of our knowledge, no other cytokines possibly released from particle-treated NR8383 cells induce cell death in L-929 cells. The rank order for this effect was Z100MP, TetricEvoCeram, Filtek Supreme and GrandiO, whereas no significant effect was found for Gradia Direct (Fig. 6). Interestingly, Z100MP and TetricEvoCeram remained, at least in part, nanosized under cell culture conditions and it may be speculated that this circumstance may be the underlying cause for the higher degree of TNF-α induction. TNF-α is one of the most important pro-inflammatory cytokines in the lungs which also plays a role in cell apoptosis or neoplastic transformation, depending e.g. on the receptor composition of recipient cells. Except for Z100 MP, the limited TNF-α induction again suggests that composite particles have no inflammatory potential up to a cellular does of 60 pg per cell.

## Conclusions

To conclude, five respirable composite dust particles were prepared and quantitatively ingested by alveolar macrophages *in vitro*. All particles led to uniform but very moderate membrane damage upon the highest concentration tested (180 μg/ml, equalling 120 pg/cell). A low inflammatory potential and some dose-dependent changes depending on the type of composite were noted with respect to H_2_O_2_ and TNF-α induction, which may be used to differentiate composite particles with respect to their bioactivity. These results are reassuring, but future research should focus on characterizing the actual exposure of dental personnel to composite dust in a very detailed way for all different types of dental treatment (restorative, orthodontic treatment, etc.). Moreover, the results of this study warrant future mechanistic research investigating possible toxic pathways.

## Additional file

Additional file 1: Uptake of composite dusts by NR8383 macrophages in F-12K medium. All photomicrographs were taken after 16 h. The left images represent light microscopic images under cell-free culture conditions at the bottom of the culture vessel, whereas the uptake of the sedimented particles by the macrophages is shown in the right images. Note that the space between the cells has been cleared from particles and most macrophages appear dark due to particle uptake, indicating that the macrophages have ingested the entire dose. (JPG 6308 kb)

## Abbreviations

DQ12: Quartz DQ12
LDH: Lactate dehydrogenase
ROS: Reactive oxygen species
TEM: Transmission electron microscopy
TNF-α: Tumor necrosis factor
