# Supplementary figures and images for: Interaction of rat alveolar macrophages with dental composite dust
Source: Part Fibre Toxicol. 2016 Nov 26;13:62. doi: 10.1186/s12989-016-0174-0 (PMC5124269; doi:10.1186/s12989-016-0174-0)

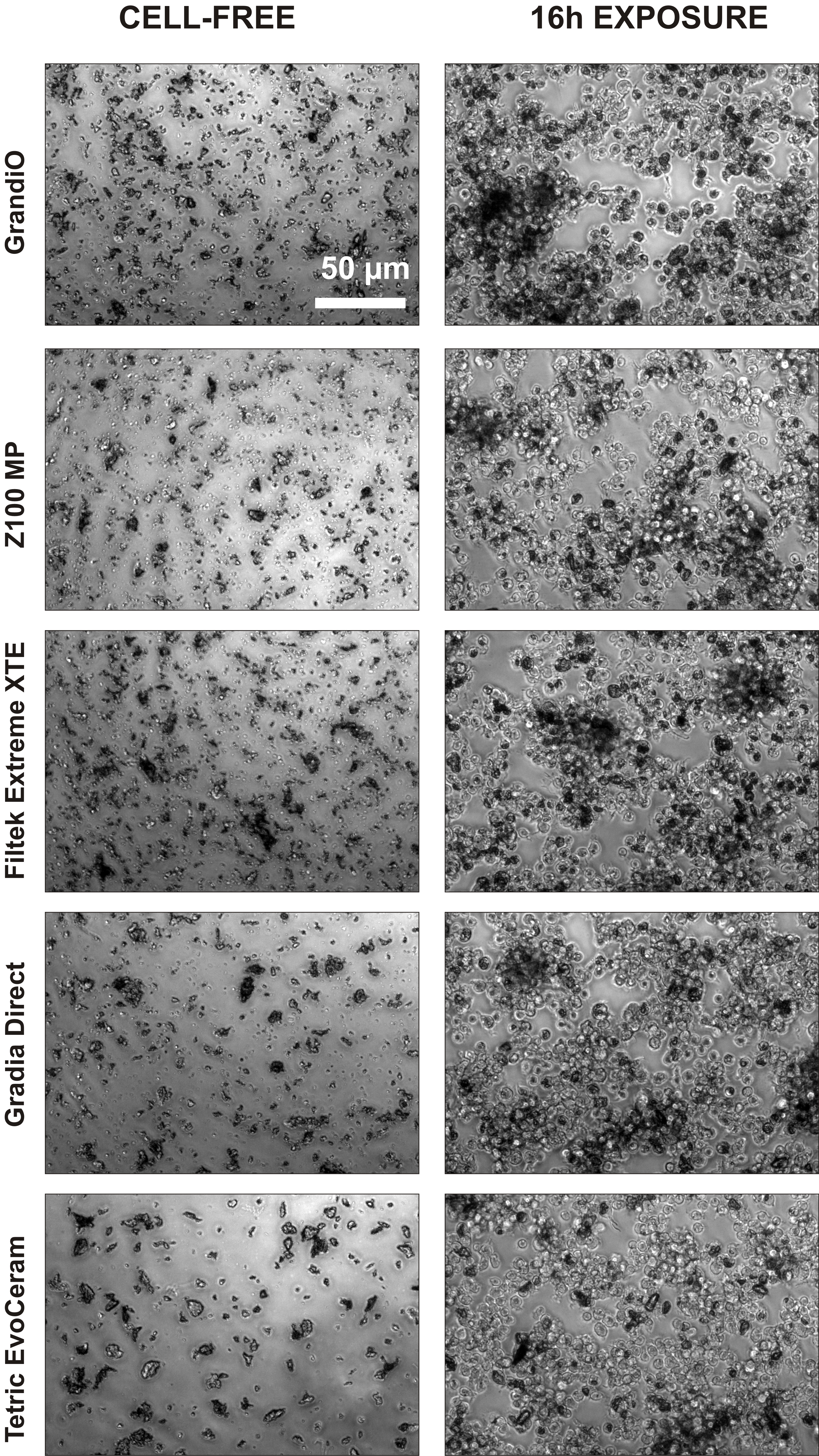

Supplement: Additional file 1: — Uptake of composite dusts by NR8383 macrophages in F-12K medium. All photomicrographs were taken after 16 h. The left images represent light microscopic images under cell-free culture conditions at the bottom of the culture vessel, whereas the uptake of the sedimented particles by the macrophages is shown in the right images. Note that the space between the cells has been cleared from particles and most macrophages appear dark due to particle uptake, indicating that the macrophages have ingested the entire dose. (JPG 6308 kb) [file 12989_2016_174_MOESM1_ESM.jpg]
